# Using graph theory to analyze biological networks

**DOI:** 10.1186/1756-0381-4-10

**Published:** 2011-04-28

**Authors:** Georgios A Pavlopoulos, Maria Secrier, Charalampos N Moschopoulos, Theodoros G Soldatos, Sophia Kossida, Jan Aerts, Reinhard Schneider, Pantelis G Bagos

**Affiliations:** 1 Department of Computer Science and Biomedical Informatics, University of Central Greece, Lamia, 35100, Greece; 2 Faculty of Engineering - ESAT/SCD, Katholieke Universiteit Leuven, Kasteelpark Arenberg 10, 3001, Leuven-Heverlee, Belgium; 3 Structural and Computational Biology Unit, EMBL, Meyerhofstrasse 1, 69117, Heidelberg, Germany; 4 Department of Computer Engineering & Informatics, University of Patras, Rio, 6500, Patras, Greece; 5 Bioinformatics & Medical Informatics Team, Biomedical Research Foundation, Academy of Athens, Soranou Efessiou 4, 11527, Athens, Greece; 6 Life Biosystems GmbH, Belfortstrasse 2, 69117, Heidelberg, Germany; 7 Luxembourg Centre for Systems Biomedicine (LCSB), University of Luxembourg, Campus Limpertsberg, 162 A, avenue de la Faïencerie, L-1511 Luxembourg

**Keywords:** biological network, clustering analysis, graph theory, node ranking

## Abstract

Understanding complex systems often requires a bottom-up analysis towards a systems biology approach. The need to investigate a system, not only as individual components but as a whole, emerges. This can be done by examining the elementary constituents individually and then how these are connected. The myriad components of a system and their interactions are best characterized as networks and they are mainly represented as graphs where thousands of nodes are connected with thousands of vertices. In this article we demonstrate approaches, models and methods from the graph theory universe and we discuss ways in which they can be used to reveal hidden properties and features of a network. This network profiling combined with knowledge extraction will help us to better understand the biological significance of the system.

## Introduction

The theory of complex networks plays an important role in a wide variety of disciplines, ranging from computer science, sociology, engineering and physics, to molecular and population biology. Within the fields of biology and medicine, potential applications of network analysis include for example drug target identification, determining a protein's or gene's function, designing effective strategies for treating various diseases or providing early diagnosis of disorders. Protein-protein interaction (PPI) networks, biochemical networks, transcriptional regulation networks, signal transduction or metabolic networks are the highlighted network categories in systems biology often sharing characteristics and properties.

**Protein-protein interaction (PPI) networks **[1] mainly hold information of how different proteins operate in coordination with others to enable the biological processes within the cell. Despite the fact that for the majority of proteins the complete sequence is already known, their molecular function is not yet fully determined. Predicting protein function is still a bottleneck in computational biology research and many experimental and computational techniques have been developed in order to infer protein function from interactions with other biomolecules. Large-scale and high-throughput techniques can detect proteins that interact within an organism. Among them, the most well-known are the pull down assays [2], tandem affinity purification (TAP) [3], yeast two-hybrid (Y2H) [4], mass spectrometry [5], microarrays [6] and phage display [7]. Some very well-known datasets that have been recently produced by employing the aforementioned techniques and that are widely used are the Tong [8], Krogan [9], DIP [10], MIPS [11], Gavin 2002 [5] and Gavin 2006 [12] datasets. Besides the various experimental methods, a variety of large biological databases that contain information concerning PPI data is already available and most of them are organism specific. Some well-known databases are the Yeast Proteome Database (YPD) [13], the Munich Information Center for Protein Sequences (MIPS) [14], the Molecular Interactions (MINT) database [15], the IntAct database [16], the Database of Interacting Proteins (DIP) [10], the Biomolecular Interaction Network Database (BIND) [17], the BioGRID database [18], the Human Protein Reference Database (HPRD) [19], the HPID [20] or the DroID [21] for Drosophila. Two additional well-documented services based on text mining analysis are the Stitch [22] and String [23] databases.

**Regulatory networks (GRNs) **contain information concerning the control of gene expression in cells. This process is modulated by many variables, such as transcription factors [24], their post-translational modifications or association with other biomolecules [25]. Usually, these networks use a directed graph representation in an effort to model the way that proteins and other biological molecules are involved in gene expression and try to imitate the series of events that take place in different stages of the process. They often exhibit specific motifs and patterns concerning their topology. Data collection, data integration and analysis techniques give now the possibility to study gene regulatory networks in a larger scale [26]. Protein-DNA interaction data is collected in databases like JASPAR [27], TRANSFAC [28,29] or B-cell interactome (BCI) [30], while post-translational modification can be found in databases like Phospho.ELM [31], NetPhorest [32] or PHOSIDA [33].

**Signal transduction networks **often use multi-edged directed graphs to represent a series of interactions between different bioentities such as proteins, chemicals or macromolecules and to investigate how signal transmission is performed either from the outside to the inside of the cell, or within the cell. Environmental parameters change the homeostasis of the cell and, depending on the circumstances, different responses can be triggered. Similarly to GRNs, these networks also exhibit common patterns and motifs concerning their topology [34]. Databases that store information about signal transduction pathways are MiST [35], TRANSPATH [36], etc.

**Metabolic and biochemical networks **[37] are powerful tools for studying and modelling metabolism in various organisms. As metabolic pathways, we consider a series of chemical reactions occurring within a cell at different time points. The main role within a metabolic network is played by the enzymes, since they are the main determinants in catalyzing biochemical reactions. Often, enzymes are dependent on other cofactors such as vitamins for proper functioning. The collection of pathways, holding information about a series of biochemical events and the way they are correlated, is called a metabolic network. Modern sequencing techniques allow the reconstruction of the network of biochemical reactions in many organisms, from bacteria to human [38,39]. Among the several databases holding information about biochemical networks some of the most popular are the Kyoto Encyclopedia of Genes and Genomes (KEGG) [40], EcoCyc [41], BioCyc [42] and metaTIGER [43]. Several methods have also been discovered to analyze the pathway structure of metabolic networks [44-48].

Many computer readable formats are available to describe biological networks. The *Systems Biology Markup Language (SBML*) [49] is an XML-like machine-readable language, that is able to represent models to be analyzed by a computer. SBML can represent metabolic networks, cell signaling pathways, regulatory networks, and many other kinds of systems [50]. Other file formats that can represent biological networks are the *Proteomics Standards Initiative Interaction (PSI-MI) *[51], *Chemical Markup Language *(*CML*) [52,53] for chemicals or *BioPAX *[54] for pathways. Secondary formats that can also be used in similar ways are the *Cell Markup Language *[55] which is an XML-like machine-readable language mainly developed for the exchange of computer-based mathematical models or the *Resource Description Framework, RDF *which is a language for the representation of information about resources on the World Wide Web [56,57].

After having given a short overview of how data can be produced either experimentally or retrieved from various databases and which formats are available for each type of network, we further emphasize on the computational analysis as defined in graph theory. We finally conclude by describing which properties of the ones discussed below characterize the various networks.

## Graph Theory and Definitions

To introduce the basic concepts of graph theory, we give both the empirical and the mathematical description of graphs that represent networks as they are originally defined in the literature [58,59].

### Undirected single graph

A graph *G *can be defined as a pair *(V, E) *where *V *is a set of vertices representing the nodes and *E *is a set of edges representing the connections between the nodes. We define as *E *= *{(i, j)| i, j *∈ *V} *the single connection between nodes *i *and *j*. In this case, we say that *i *and *j *are ***neighbors***. A *multi-edge connection *consists of two or more edges that have the same endpoints. Such multi-edges are especially important for networks in which two elements can be linked by more than one connection. In such cases, each connection indicates a different type of information. This is an important feature since there are networks such as protein-protein interaction networks in which two proteins might be evolutionary related, co-occur in the literature or co-express in some experiments, resulting by this way in three different connections, each one with a different meaning. An example of PPI database that takes into account the different types of interactions between proteins is String [23].

### Directed graph

A directed graph is defined as an ordered triple *G *= *(V, E, f)*, where *f *is a function that maps each element in *E *to an ordered pair of vertices in *V*. The ordered pairs of vertices are called ***directed edges, arcs or arrows***. An edge *E *= *(i, j) *is considered to have direction from ***i ***to ***j***. Directed graphs are mostly suitable for the representation of schemas describing biological pathways or procedures which show the sequential interaction of elements at one or multiple time points and the flow of information throughout the network. These are mainly metabolic, signal transduction or regulatory networks [34].

### Weighted graph

A weighted graph is defined as a graph *G = (V, E) *where *V *is a set of vertices and *E *is a set of edges between the vertices *E = {(u, v) | u, v *∈ *V} *associated with it a weight function *w: E→R*, where *R *denotes the set of all real numbers. Most of the times, the weight *w_ij _*of the edge between nodes *i *and *j *represents the relevance of the connection. Usually, a larger weight corresponds to higher reliability of a connection. Weighted graphs are currently the most widely used networks throughout the field of bioinformatics. As an example, relations whose importance varies are frequently assigned to biological data to capture the relevance of co-occurrences identified by text mining, sequence or structural similarities between proteins or co-expression of genes [23,60].

***Bipartite graph ***is an undirected graph *G *= (*V, E*) in which *V *can be partitioned into 2 sets *V*_1 _and *V*_2 _such that (*u*,*v*) ∈ *E *implies either *u *∈ *V*_1 _and *v *∈ *V*_2 _OR *v *∈ *V*_1 _and *u *∈ *V*_2_. Applications of this type of graph to visualization or modeling of biological networks range from representation of enzyme-reaction links in metabolic pathways to ontologies or ecological connections, as discussed in [61] or [62].

If *G = (V, E) *is a graph, then *G_1 _*= *(V_1_, E_1_) *is called a ***subgraph ***or if *V_1 _⊆ V *and *E_1 _⊆ E*, where each edge in *E_1 _*is incident with vertices in *V_1_*.

**Figure 1:**
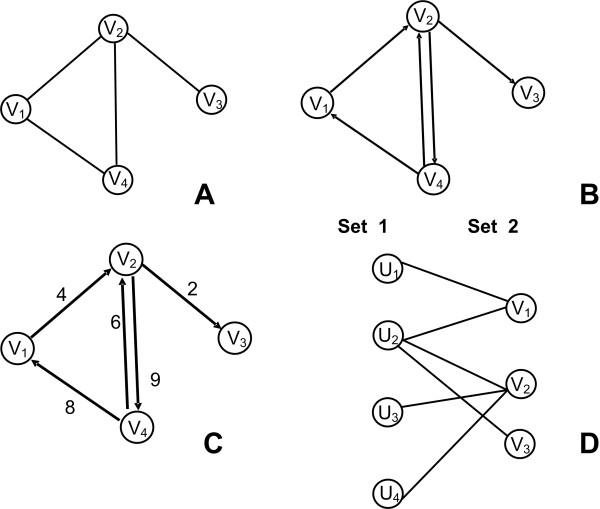
**Undirected, Directed, Weighted, Bipartite graphs**. A. Undirected Graph: *V = {V_1_, V_2_, V_3_, V_4_}, |V| = 4, E = {(V_1_, V_2_), (V_2_, V_3_), (V_2_, V_4_), (V_4_, V_1_)}, |E| = 4*. B. Directed Graph: *V *= *{V_1_, V_2_, V_3_, V_4_}, |V| = 4, E = {(V_1_, V_2_), (V_2_, V_3_), (V_2_, V_4_), (V_4_, V_1_), (V_4_, V_2_)}, |E| = 5*. C. Weighted Graph: *V = {V_1_, V_2_, V_3_, V_4_}, |V| = 4, E = {(V_1_, V_2_, V_4_), (V_2_, V_3_, V_2_), (V_2_, V_4_, V_9_), (V_4_, V_1_, V_8_), (V_4_, V_2_, V_6_)}, |E| = 5*. D. Bipartite graph: *V *= *{U_1_, U_2_, U_3_, U_4_, V_1_, V_2_, V_3_}, |V| = 7, E = {(U_1_, V_1_), (U_2_, V_1_), (U_2_, V_2_), (U_2_, V_3_), (U_3_, V_2_), (U_4_, V_2_)}, |E| = 6*.

Examples and shapes describing the aforementioned graph types can be found in Figure 1. The most common data structures that are used to make these networks computer readable are adjacency matrices or adjacency lists. The following section provides a short mathematical description of these data structures.

The ***degree of a node ***in an undirected graph is the number of connections or edges the node has to other nodes and is defined as *deg(i) = k(i) = |N(i)| *where *N(i) *is the number of the neighbors of node *i*. If a network is directed, then each node has two different degrees, the ***in-degree ****deg*_*in *_*(i) *which is the number of incoming edges to node *i*, and the ***out-degree ****deg*_*out*_*(i) *which is the number of outgoing edges from node *i*. The ***total connectivity ***of a network is defined as  where *E *is the number of edges and *N *the total number of nodes. The connectivity structure of biological networks is often informative with respect to reaction interplay and reversibility, compounds that structure the network, like in metabolism, or trophic relationships, like in food-web networks. Such connectivity profiles can be detected based on mixture models using software like MixNet [63].

## Data Structures

The two main data structures used to store network graph representations are described below.

### Adjacency matrix

Given a graph *G *= *(V, E) *the adjacency matrix representation consists of a *|V|x|V| *= *nxn *matrix *A *= *(a_ij_) *such that *a_ij _= 1 *if *(i, j)*∈*V *or *a_ij _= 0 *or otherwise , *n *= |*V*|. In the case where we have weighted graphs *a_ij _= w_ij _if (i, j)*∈*V *or *a_ij _= 0 *otherwise. For **undirected graphs **the matrix is symmetric because *a_ij _= a_ji_*. The aforementioned rule does not apply to directed graphs, because in that case the upper and the lower triangle parts of the matrix reveal the direction of the edges. Adjacency matrices require space of *Θ(|V|^2^) *and are best suited for dense and not for sparse graphs. For an all-against-all symmetric data set, only the upper or the lower triangular part of the matrix is necessary, which requires *Θ(|V|) *amount of memory to be allocated. This data structure is more efficient for cluttered networks, where the density of the connections between elements is relatively high. In the case of a fully connected graph where all nodes are connected with each other, adjacency matrices are highly suggested. To reduce memory allocation to half for larger scale data, a symmetric *2D *matrix *A *can be stored as a *1D *matrix *B*, where  if the first element is α_11 _like for example in Matlab platform or  if the first element is α_00 _like in most programing languages. Matrix *B *currently hosts the lower part of matrix *A*. If for example *A *is a 3 × 3 matrix starting from element α_11_,  then matrix *B *is defined as *B *= *{α_11_,α_21,_α_22_,α_31_,α_32_,α_33_}*. The 1D array will be of size  including the diagonal.

### Adjacency list

**Figure 2:**
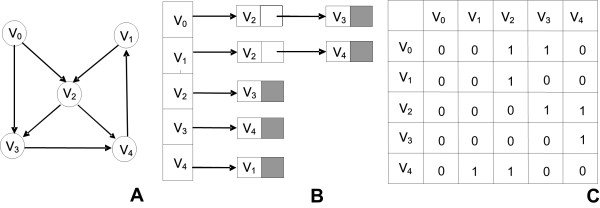
**Data structures**. A. A Directed Graph: A random graph consisting of five nodes and six directed edges. B. Adjacency List: The data structure which represents the directed graph using lists. C. Adjacency Matrix: The data structure which represents the directed graph using a 2D matrix. The zeros represent the absence of the connection whereas the ones represent the existence of the connection between two nodes. The matrix is not symmetric since the graph is directed.

Given a graph *G *= *(V, E) *the adjacency list representation consists of an array *Adj *of *|E| *elements where for each *e*∈*E Adj(0, e) = i *∈*V*. Adjacency lists require space *Θ (|V|+|E|) *and are preferable for sparse graphs with a low density of connections. An example of how these data structures represent a graph is given in Figure 2.

## Network Properties

Looking at different network properties can provide valuable insight into the internal organization of a biological network, the repartition of molecules among cellular processes, as well as the evolutionary constraints that have shaped an organism's protein, metabolic or regulatory network into a functional, feasible structure. In the following, we give a short description of the main properties that are commonly analyzed in networks.

The ***graph density ***shows how sparse or dense a graph is according to the number of connections per node set and is defined as . A ***sparse graph ***is a graph where *|E| *= *O(|V|^k^*) and *2 > k > 1 *or otherwise when *|E| " |V|*. ***Dense ***is a graph where *|E| " |V|^2^*. It has been argued that biological networks are generally sparsely connected, as this confers an evolutionary advantage for preserving robustness. This has been observed for a series of organisms: the transcriptional regulatory networks of *S. cerevisiae*, *E. coli*, *D. melanogaster *all have connectivity densities lower than 0.1 [64].

In the mathematical field of graph theory, a ***complete graph ***is a simple graph in which every pair of distinct vertices is connected by a unique edge. The complete graph on *n *vertices has , *n* = *|V| *number of edges and it is a regular graph of degree *|V| **- 1***.

### Graph Isomorphism

**Figure 3:**
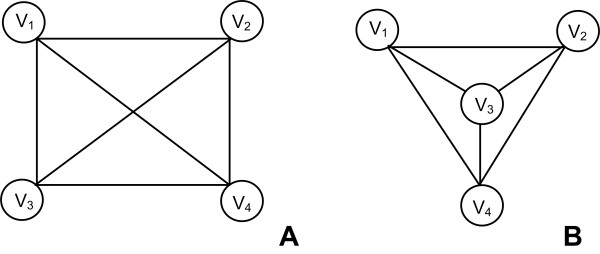
**Graph Isomorphism**. *V = {V_1_, V_2_, V_3_, V_4_}, |V| = 4, E = {(V_1_, V_2_), (V_1_, V_3_), (V_1_, V_4_), (V_2_, V_3_), (V_2_, V_4_), (V_3_, V_4_)}, |E| = 6*. Graphs *A *and *B *have different topology but they are isomorphs. The graph is fully connected and every node is connected to any other so that it forms a fully connected clique.

Let *G*_*1*_= (*V*_*1*_, *E*_*1*_) and *G*_*2*_= (*V*_*2*_, *E*_*2*_) be two undirected graphs. A function *f*: *V*_*1 *_->*V*_*2 *_is called isomorphism if *f *is an edge-preserving bisection, such that for all *a, b*∈*V*_*1*_, *(a, b)*∈*E*_*1 *_if and only if *(f(a), f(b)) *∈ *E*_*2*_. When such function exists, then *G*_*1 *_and *G*_*2 *_are called isomorphic. An example is shown in Figure 3.

**Figure 4:**
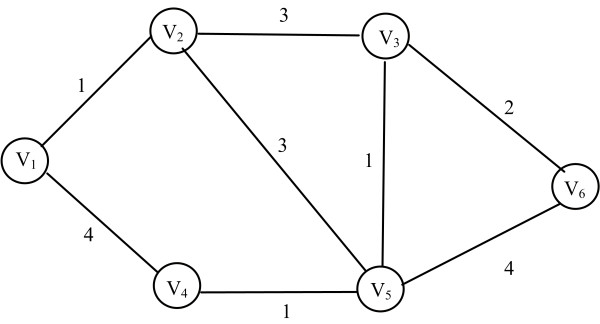
**Walks, simple paths trails and cycles in graphs**. A *walk *is a sequence of nodes *e.g*. (*V_2_, V_3_, V_6_, V_5_, V_3_*). A *simple path *is a walk with no repeated nodes, e.g. (*V_1_, V_4_, V_5_, V_2_, V_3_*). A trail is a walk where no edges are repeated e.g. (*V_1_, V_2_, V_3 _V_6_*). A *cycle *is a walk (*V_1_, V_2_,..., V_L_*) where *V_1 _= V_L _*with no other nodes repeated and *L*>3, e.g. (*V_1_, V_2_, V_5_, V_4_, V_1_*).

A ***walk ***is a pass through a specific sequence of nodes *(v_1_, v_2_,..., v_L_) *such that *{(v_1_, v_2_), (v_2_, v_3_),..., (v_L-1_, v_L_)} ⊆ E*. A ***simple path ***is a walk with no repeated nodes. A ***cycle ***is a walk *(v_1_, v_2_,..., v_L_) *where *v_1 _= v_L _*with no other nodes repeated and *L *>*3*, such that the last node is the same with the first one. A ***trail ***is a path where no edge can be repeated. A graph is called ***cyclic ***if it contains a cycle. In any other case it is called ***acyclic***. All of the aforementioned can be found as an example in Figure 4. A ***complete graph ***is a graph in which every pair of nodes is ***adjacent***. If *(i, j) *is an edge in a graph *G *between nodes *i *and *j*, we say that the vertex *i *is *adjacent *to the vertex *j*. An undirected graph is ***connected ***if one can get from any node to any other node by following a sequence of edges. A directed graph is ***strongly connected ***if there is a directed path from any node to any other node. This does not require an all-against combination. The ***distance ****δ(i, j) *from *i *to *j *is the length of the *shortest path *from *i *to *j *in *G*. If no such path exists, then we set *δ*(*i, j*) = *∞ *assuming that the nodes are so far between each other so they are not connected. Practically, for the distance *δ*(*i, j*) = *∞ *we can use the maximum weight of the graph by adding one. Thus *δ(i, j) *= ∞ = *(max_d(i, j)_+1)*. To define the shortest path problem we can briefly say that it is the methodology of finding a path between two nodes such that the sum of the weights of its constituent edges is minimized. The ***average path length ***and the ***diameter ***of a graph *G *are defined to be the average and maximum value of *δ*(*i, j*) taken over all pairs of distinct nodes, *i, j *∈*V*(*G*) which are connected by at least one path. More specifically, the average path length of a network is the average number of edges or connections between nodes, which must be crossed in the shortest path between any two nodes. It is calculated as  where *δ_min_(i, j) *is the minimum distance between nodes *i *and *j*. The diameter of a network is the longest shortest path within a network. The ***diameter ***is defined as . The most common algorithms for calculating the shortest paths are ***Dijkstra***'s greedy algorithm [65] and ***Floyd's ***dynamic algorithm [66]. *Dijkstra's *algorithm has running time complexity *O(N^2^) *where *N *is the number of vertices and returns the shortest path between a source vertex *i *and all other vertices in the network. *Floyd's *algorithm has running time complexity *O(N^3^) *and requires an all-against-all matrix that contains the distances of every node in the network to every other node in the network.

A ***clique ***in an undirected graph *G *is a subgraph *G' *which is complete. An independent set in a graph is a subset of the vertices such that no pair of vertices is an edge in the graph. The size of a clique comes from the number of vertices it contains. A ***maximal clique ***is a clique that cannot be extended by including one more adjacent vertex, i.e. a clique which does not exist exclusively within the vertex set of a larger clique. A maximum clique is a clique of the largest possible size in a given graph. The clique problem refers to the problem of finding the largest clique in any graph *G*. This problem is *NP*-complete, and as such, many consider that it is unlikely that an efficient algorithm for finding the largest clique of a graph exists. Figure 3b shows a clique. A very famous method to find maximal cliques in a graph is the so-called Bron-Kerbosch algorithm [67]. Detection and analysis of these structures has found many biological applications: identifying groups of consistently co-expressed genes in microarray datasets, finding cis regulatory motifs or matching three-dimensional structures of molecules [68,69]. Several tools have been developed for clique identification, like Clique Finder within the Arabidopsis Co-expression Tool server [70] or MIClique [68]. Bioconductor [71] provides a large collection of software for clique analysis.

**Figure 5:**
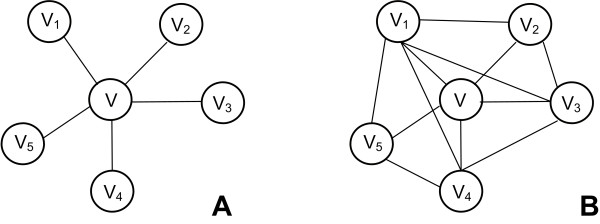
**Clustering Coefficient**. A) Node *V *behaves like a hub but it has clustering coefficient *C = 0*. B) Node *V *comes with a high clustering coefficient. The maximum number of potential connection is given by *E_max_=|V|(|V|-1)/2 *where *|V| = 5 *is the number of the neighbors of node V, thus *E_max _= 10*. The neighbors of node V are connected with 7 edges between each other, *E *= *{(V_1_, V_2_), (V_2_, V_3_), (V_3_, V_4_), (V_4_, V_5_), (V_5_, V_1_), (V_1_, V_3_), (V_1_, V_4_)}*. The clustering coefficient of node V is *C *= *E_V_/E_max _= 7/10 = 0.7*.

***Clustering Coefficient ***is the measurement that shows the tendency of a graph to be divided into clusters. A cluster is a subset of vertices that contains lots of edges connecting these vertices to each other. Assuming that *i *is a vertex with degree *deg(i) = k *in an undirected graph *G *and that there are *e *edges between the *k *neighbors of *i *in *G*, then the *Local Clustering Coefficient *of *i *in *G *is given by . Thus, *C_i _*measures the ratio of the number of edges between the neighbors of *i *to the total possible number of such edges, which is *k*(*k-*1)*/*2. It takes values as 0 ≤ *C_i _*≤ 1. The ***average Clustering Coefficient ***of the whole network *C_average _*is given by  where *N=|V| *is the number of vertices. The closer the local clustering coefficient is to 1, the more likely it is for the network to form clusters. Obviously, a clique would come with local clustering coefficient equal to 1. An example showing how local clustering coefficient is calculated is shown in Figure 5.

Biological networks have a significantly higher average clustering coefficient compared to random networks, which proves their modular nature. Indeed, many cellular processes are governed by subsets of biomolecules that form an interaction module. Since cellular processes are linked, the modules tend to be linked as well, but the linking molecules are often few, such that the module overlap is quite low [72,73].

***Centralization ***is the measurement that shows whether a network has a star-like topology or whether the nodes of the network have on average the same connectivity. The closer the centralization is to 1, the more likely is the network to have a star-like topology. The closer to 0, the more likely it is that the nodes of the network have on average the same connectivity (for example a square, where every node is connected with 2 neighbors). It is calculated as

**Figure 6:**
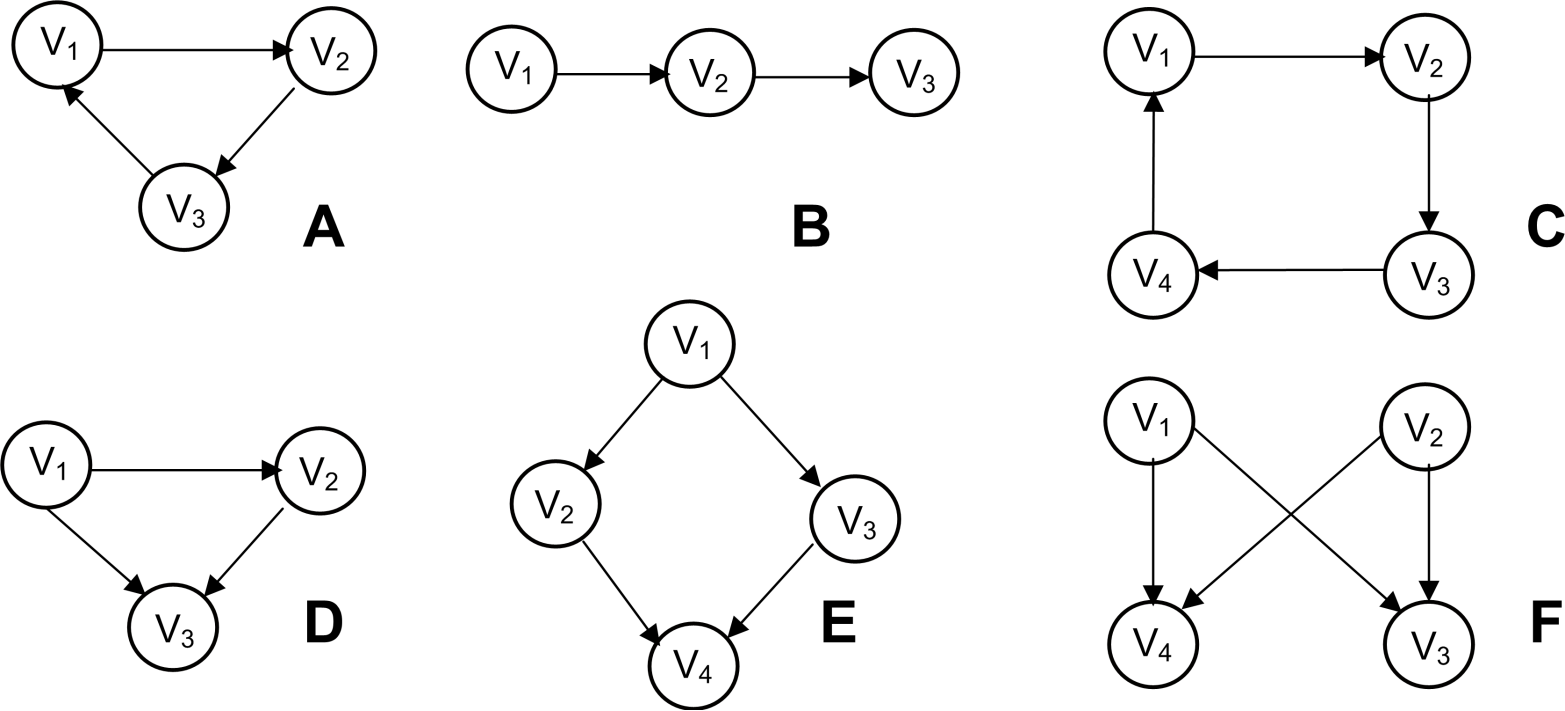
**Network Motifs**. Some common network motifs. A) *Feed-forward loop*. Type of networks: protein, neuron, electronic. B) *Three chain*. Type of network: food webs. C) *Four node feedback*. Type of network: gene regulatory, electronic. D) *Three node feedback*. Type of network: gene regulatory, electronic. E) *Bi-parallel*. Type of network: gene regulatory, biochemical. F) *Bi-Fan*. Type of networks: protein, neuron, electronic [74].

***Network Motifs ***represent patterns in complex networks occurring significantly more often than in randomized networks [74]. They consist of subgraphs of local interconnections between network elements. A motif is a small connected graph *G'*. A *match G' *of a motif in graph *G *is a graph *G'' which *is isomorphic to *G' *and a subgraph of *G*. Signal transduction and gene regulatory networks tend to be described by various motifs [72,75]. Although motif determination gives lots of information concerning the properties and the characteristics of a network, it does not necessarily reveal evidence about its function and the function of its components [76]. However, some motifs have been found to be associated with optimized biological functions, like in the case of positive and negative feedback loops, oscillators or bifans [73]. Figure 6 shows the most common motifs that are found in various networks.

## Network Centralities and Node Ranking

This section shows how nodes can be ranked or sorted according to their properties, depending on the question asked. In biological networks, it is important for example to detect central nodes or intermediate nodes that affect the topology of the network, depending of course on the biological question. Such a question would be to find the molecules in a biological pathway that are not necessarily central but have a crucial biological role in signal transduction or in PPI networks, to detect such nodes that interact with many other proteins or find molecules that are crucial for stimulating the expression of genes.

***Degree Centrality ***shows that an important node is involved in a large number of interactions. For a node *i*, the degree centrality is calculated as *C_d_(i) *= *deg(i)*. For directed graphs, each node is obviously characterized by two degree centralities. These are *C_d in_(i) *= *deg_in_(i) and C_d out_(i) *= *deg_out_(i)*. Nodes with very high degree centrality are called ***hubs ***since they are connected to many neighbors (see Figure 5). Scale-free networks tend to contain hubs. The removal of such central nodes has great impact on the topology of the network. It has been shown that biological networks tend to be robust against random perturbations, but disruption of hubs often leads to system failure [77,78].

**Figure 7:**
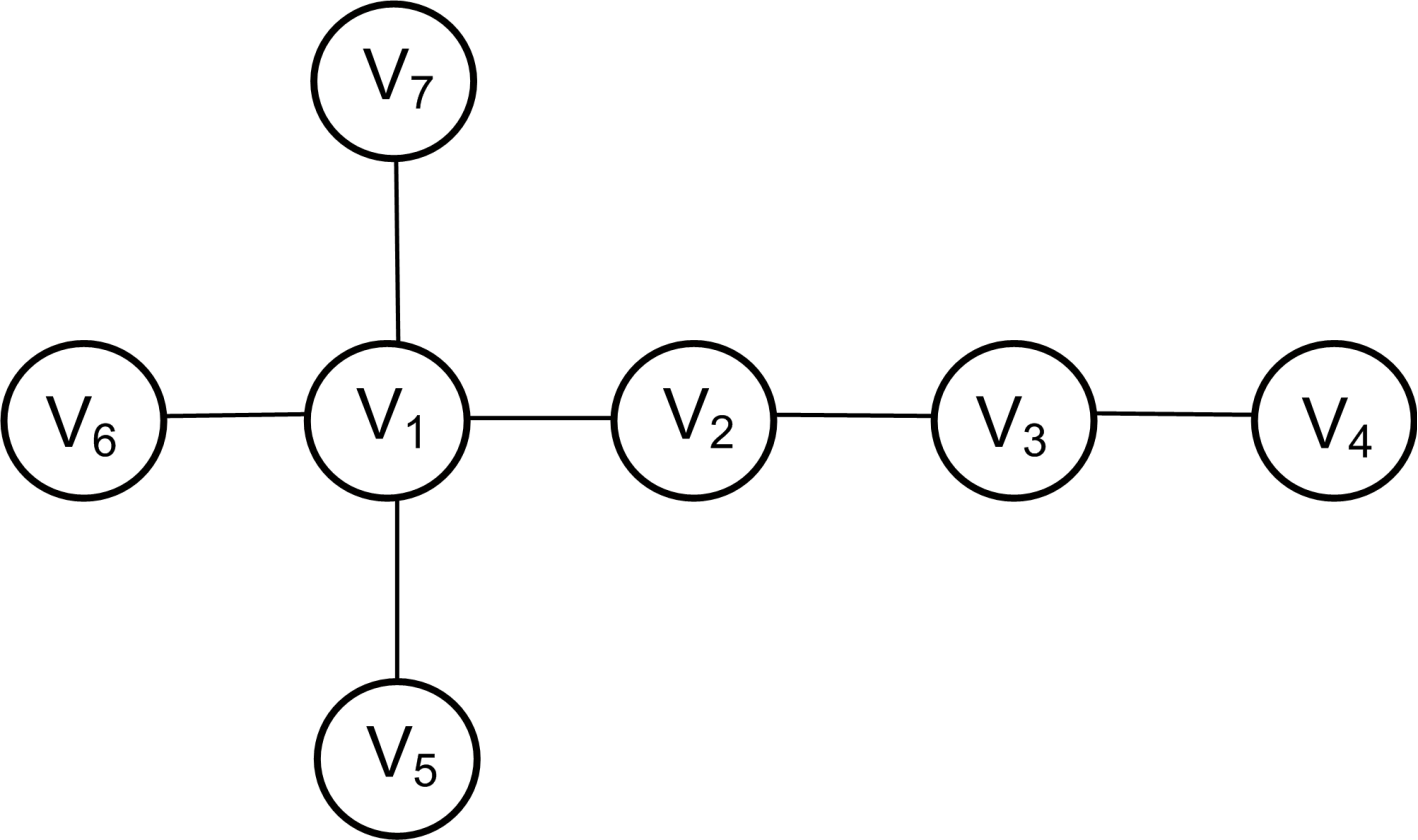
**Closeness and Betweeness centralities**. **Closeness centrality. *V_1_***: d_1 _= 4 × 1 + 1 × 2 + 1 × 3 = 9, *C_clo_(1) *= 6/9. *V_1 _*accesses 4 nodes *(V_2_, V_5_, V_6_, V_7_) *with step 1, 1 node *(V_3_) *with step 2 and 1 node *(V_4_) *with step 3. 6 nodes can be accessed in total by V_1_. ***V_2_***: d_2 _= 2 × 1 + 4 × 2 = 10 > d_1_, *C_clo_(2) *= 6/10. *V_2 _*accesses 2 nodes *(V_1_, V_3_) *with step 1 and 4 nodes *(V_4_, V_5_, V_6_, V_7_) *with step 2. 6 nodes can also be accessed in total by V_2_. As a result, V_1 _is more central than node V_2 _since d1>d_2_. Betweenness centrality. *N_p_(1) *= 12 shortest paths that pass through node *V_1_*. The paths from the starting to the ending node are *{V_2_-V_5_, V_2_-V_6_, V_2_-V_7_, V_3_-V_5_, V_3_-V_6_, V_3_-V_7_, V_4_-V_5_, V_4_-V_6_, V_4_-V_7_, V_5_-V_6_, V_5_-V_7_, V_6_-V_7_}. N_p_(2) *= 8 shortest paths that pass through node *V_2_*. The paths are {*V_1_-V_3_, V_1_-V_4_, V_3_-V_5_, V_3_-V_6_, V_3_-V_7_, V_4_-V_5_, V_4_-V_6_, V_4_-V_7_}. N_p_(3) = 5 {V_1_-V_4_, V_2_-V_4_, V_4_-V_5_, V_4_-V_6_, V_4_-V_7_}. N_p_(4) *= *N_p_(5) *= *N_p_(6) *= *N_p_(7) = 0. N_p _*= 25 the total sum of shortest paths that pass through the nodes, thus *N_p_= N_p_(1)+N_p_(2)+N_p_(3)+N_p_(4)+N_p_(5)+N_p_(6)+N_p_(7)*. The centralities are *C_b _(1) = 12/25 = 0.48, C_b _(2) = 8/25 = 0.32, C_b _(3) = 5/25 = 0.20, C_b _(4) = C_b _(5) = C_b _(6) = C_b _(7) = 0*, thus node *V_1 _*is more central.

***Closeness Centrality ***indicates important nodes that can communicate quickly with other nodes of the network. Let *G = (V, E) *be an undirected graph. Then, the centrality is defined as  where *dist(i, j) *denotes the distance or else the shortest path *p *between the nodes *i *and *j*. An example is shown in Figure 7. Closeness centrality has been used to identify the top central metabolites in genome-based large-scale metabolic networks [79], to compare unicellular and multicellular eukarya, to rank pathways and obtain a perspective on the evolution of metabolic organization [80]. A decrease in closeness centrality of components has been observed as a consequence of increased distance between pathways throughout evolution [80]. It has been chosen as the best centrality measure that can be used extract the metabolic core of a network [81].

***Betweenness Centrality ***shows that nodes which are intermediate between neighbors rank higher. Without these nodes, there would be no way for two neighbors to communicate with each other. Thus, *betweenness centrality *shows important nodes that lie on a high proportion of paths between other nodes in the network. For distinct nodes *i, j, w *∈ *V*(*G*), let *σ_ij _*be the total number of shortest paths between *i *and *j *and *σ_ij_*(*w*) be the number of shortest paths from *i *to *j *that pass through *w*. Moreover, for *w *∈ *V*(*G*), let *V *(*i*) denote the set of all ordered pairs, (*i, j*) in *V*(*G*) × *V*(*G*) such that *i, j, w *are all distinct. Then, the Betweenness Centrality is calculated as . An example is shown in Figure 7. Proteins with high betweenness centralities have been termed "bottlenecks", for their role as key connector proteins with essential functional and dynamic properties [73], for example metabolites that control the flux between two big metabolic modules. Calculation of this centrality measure is discussed in [82] and [83] and their properties within the PPI network of yeast are detailed in [84].

***Eigenvector Centrality ***ranks higher the nodes that are connected to important neighbors. Let *G = (V, E) *be an undirected graph and *A *the adjacency matrix of network *G*. The eigenvector centrality is the eigenvector *C_eiv- _*of the largest eigenvalue *λ_max _*in absolute value such that *λC_eiv _*= *AC_eiv_*. Formally, if *A *is the adjacency matrix of a network *G *with *V*(*G*) = *{v*_1_,..., *v_n_}*, and , then the eigenvector centrality *C_eiv_(v_i_) *of the node v_i _is given by the *i^th ^*coordinate *x*_*i *_of a normalized eigenvector that satisfies the condition *Ax=ρ(A)x*. Such algorithms can be used for efficient page ranking on the web. In biology this centrality measurement has been used, among others, to identify synthetic genetic interactions [85], gene-disease associations [86] or network hubs [77].

**Figure 8:**
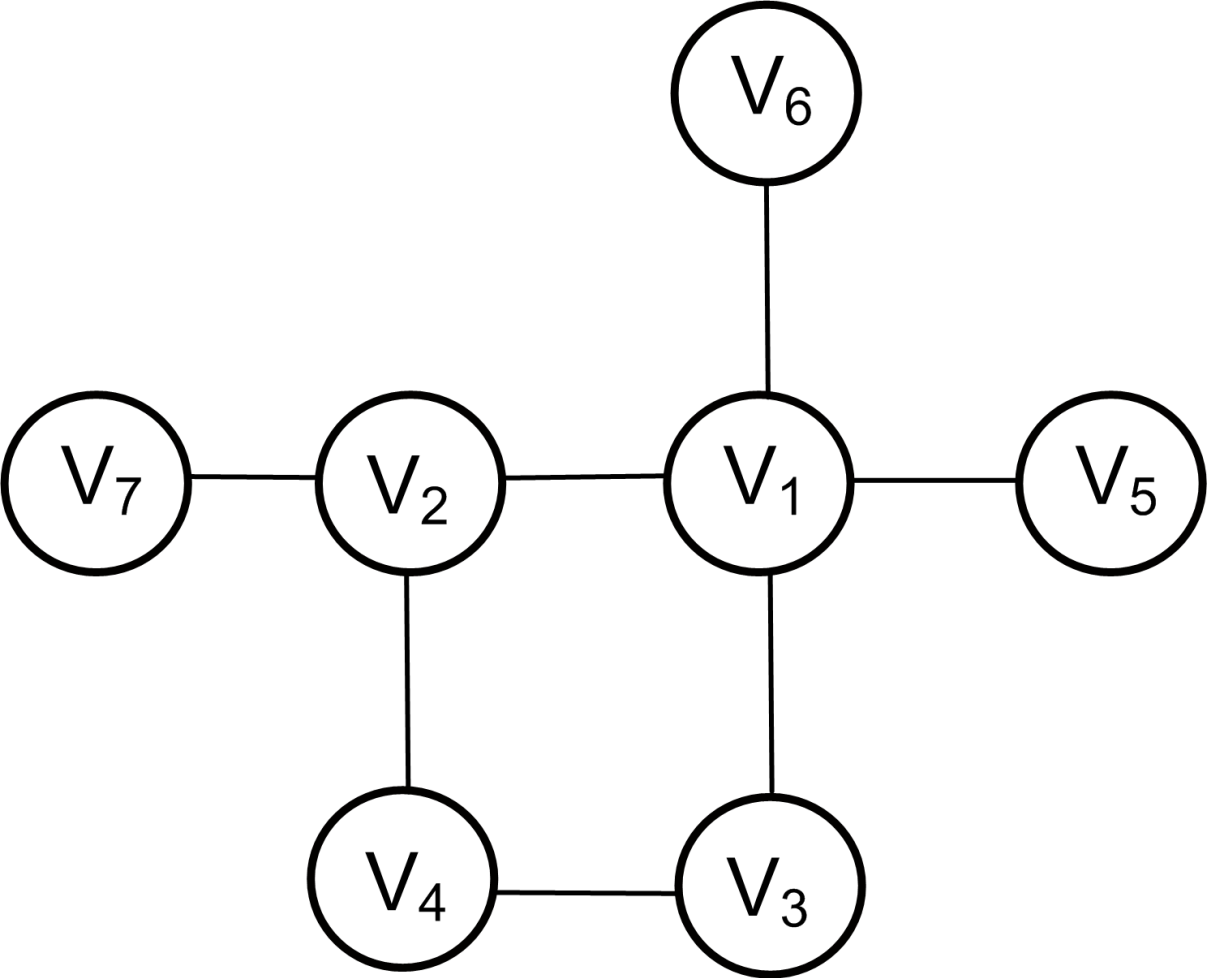
**Eccentricity Centrality**. ***V***_***1***_: 4 × 1, 2 × 2; *V*_*1 *_accesses 4 nodes *(V_2_, V_3_, V_5_, V_6_) *with step 1 and 2 nodes *(V_4_, V_7_) *with step 2. The step represents the shortest path. The maximum shortest path *d_max _*= 2. ***V***_***2***_: 3 × 1, 3 × 2; Similarly *V*_*2 *_accesses 3 nodes *(V_4_, V_7_, V_1_) *with step 1 and 3 nodes *(V_3_, V_5_, V_6_) *with step 2. The maximum shortest path *d_max _= 2*. ***V***_***3***_: 2 × 1, 3 × 2, 1 × 3; Similarly *V*_*3 *_accesses 2 nodes *(V_1_, V_4_) *with step 1, 3 nodes *(V_2_, V_5_, V_6_) *and one node *(V_7_) *with step 3. The maximum shortest path *d_max _= 3*. ***V***_***4***_: 2 × 1, 2 × 2, 2 × 3; The maximum shortest path *d*_*max*_=*3*. ***V***_***5***_: 1 × 1, 3 × 2, 2 × 3; The maximum shortest path *d*_*max *_= *3*. ***V***_***6***_: 1 × 1, 3 × 2, 2 × 3; The maximum shortest path *d*_*max *_= *3*. ***V***_***7***_: 1 × 1, 2 × 2, 3 × 3; The maximum shortest path *d*_*max *_= *3*. As a result, the ordering of the nodes according to *C*_*ecc *_: (*V*_*1*_,*V*_*2*_), (*V*_*3*_,*V*_*4*_,*V*_*5*_,*V*_*6*_,*V*_*7*_).

***Eccentricity Centrality ***is the measure that shows how easily accessible a node is from other nodes. Let *G = (V, E) *be an undirected graph. The eccentricity centrality is calculated as  where *dist(i, j) *is the shortest path between nodes *i *and *j*. The eccentricity *C_ecc _*of a vertex *V *is the greatest distance between v and any other vertex. An example is shown in Figure 8. In biological networks, proteins or other bioentities with high eccentricity are easily functionally reachable by other components of the network, and thus can readily perceive changes in concentration of other enzymes or molecules they are linked to. In contrast, those proteins that have lower eccentricities will often play a marginal functional role in the system [87].

***Subgraph Centrality ***is the measure that ranks nodes according to the number of subgraphs of the overall network in which the node participates, with more weight given to small subgraphs. Let *G = (V, E) *be an undirected graph and *A *the adjacency matrix of network *G*. The subgraph centrality of a node is calculated as . Subgraph centrality analysis has been used to study essential proteins in proteomic maps [77], to compute the degree of folding of protein chains [88], to understand the molecular structure of drug-like compounds [89] or to zoom into the topological environment of certain nodes in PPI networks of several organisms [90].

**Figure 9:**
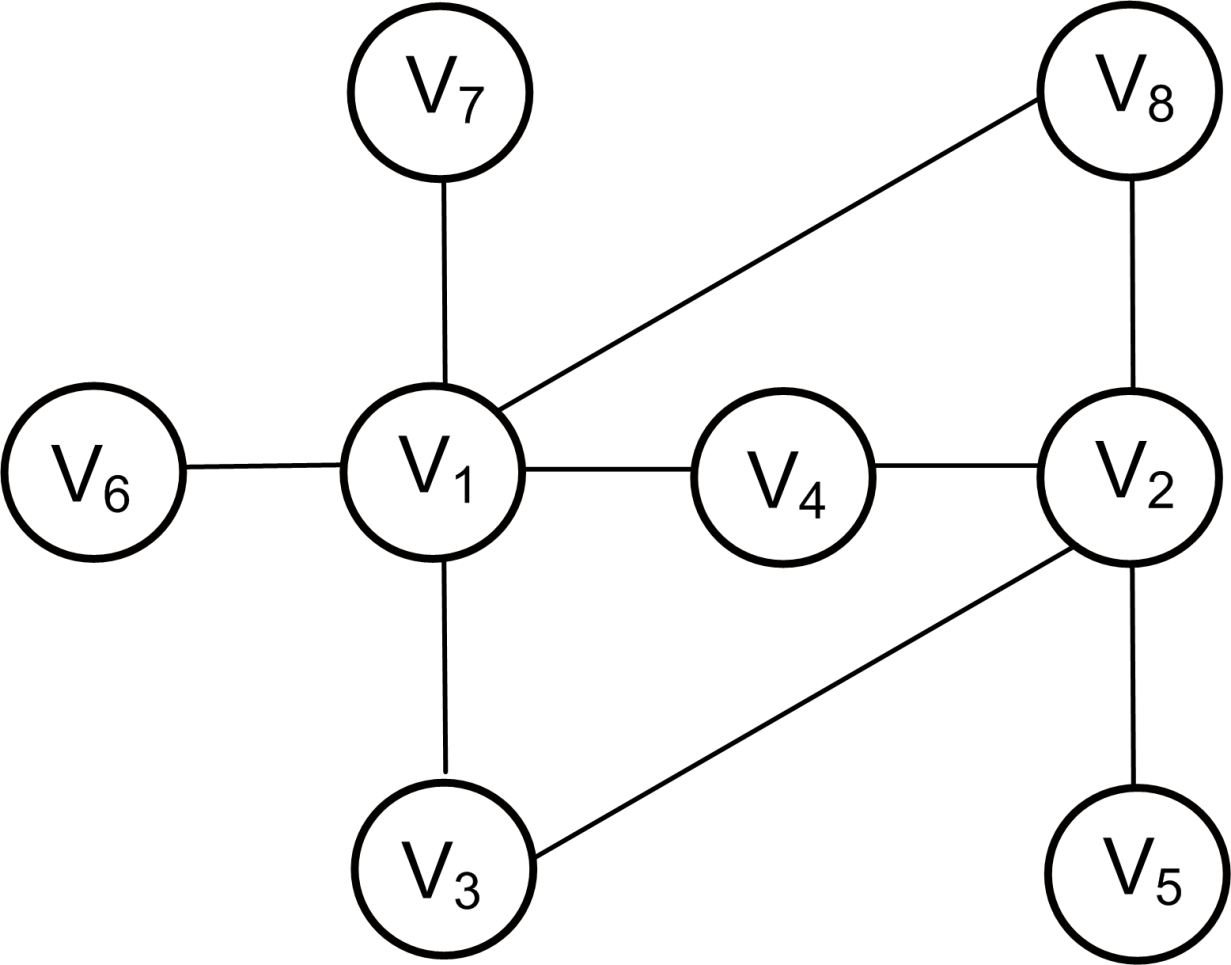
**Matching Index**. ***V_1 _***is connected with 5 nodes *(V_3_, V_4_, V_6_, V_7,_V_8_)*. ***V_2 _***is connected with 4 nodes *(V_3_, V_4_, V_5_, V_8_)*. ***V_3 _***is connected with 2 nodes *(V_1_, V_2_)*. ***V_4 _***is connected with 3 nodes *(V_1_, V_2_)*. ***V_5 _***is connected with 1 node *(V_2_)*. ***V_6 _***is connected with 1 node *(V_1_)*. ***V_7 _***is connected with 1 node *(V_1_)*. ***V_8 _***is connected with 2 nodes *(V_1_, V_5_)*. Node *V_1 _*and *V_2 _*are connected with 3 common nodes *(V_3_, V_4_, V_8_)*and in total with 6 distinct neighbors *(V_3_, V_4_, V_8_, V_5_, V_6 _, V_7_)*. The matching index will then be M_1,2 _= 3/6 = 0.5, thus *V_1 _*and *V_2 _*are functionally similar even though they are not connected.

***Matching Index ***is the measure that shows how similar two nodes are within the network. Two vertices that are functionally similar do not always have to be connected. The matching index *M*_*ij *_measures the "similarity" of two nodes and is based on the number of common neighbors shared by nodes *i *and *j*. It is calculated as

 or . An example is shown in Figure 9. The matching index is often used to cluster different components of a biological network according to some property. For instance, it has been used to describe spatial growth in brain networks during development [91] or to predict the connectivity of primate cortical networks [92].

Further centrality measurements and their application to the study of PPIs in yeast are introduced in [85]. A discussion about how centrality correlates with lethality in biological networks can be found in [93]. The coupling between centrality and essentiality has also been investigated in several eukaryotic protein networks [94]. It is very often the case that studies of a particular network involve the analysis and comparison of several centrality measures, for instance to study pleiotropy in human genetic diseases [87], to compare PPI and transcriptional regulation networks [95] or to test hub essentiality [77]. Tools that have implemented functionality for exploring the different types of centralities previously mentioned in biological networks and not only are CentiBiN [96], Visone [97], Pajek [98], VisANT [99]. In most of the cases, however, only a limited selection of centrality measures is available.

## Network Topology

The topology of the network often reveals information about its biological significance. Often, networks follow patterns and rules and have a specific topology that allows scientists to go through a deeper investigation towards knowledge extraction.

***Scale-free ***or otherwise real world networks describe natural networks like online communities (i.e Facebook) where the nodes are the people and the edges the connection between them, or networks such as the World Wide Web (www) where the nodes are individual web pages and the links are hyperlinks. Many biological networks also have scale-free properties, with nodes representing bioentities and edges the interactions between them (like proteins that interact physically or metabolites that take part in the same reaction) [73,93,100]. Assuming that *k *is the number of links originating from a given node and *P(k) *the probability that the degree of a randomly chosen vertex equals *k*, a scale-free network exhibits a power law distribution *P*(*k*) ~ *k*^-γ ^where *γ *denotes the ***degree exponent***. A scale-free network can be constructed by progressively adding nodes to an existing network and introducing links to existing nodes with preferential attachment so that the probability of linking to a given node *i *is proportional to the number of existing links *k_i _*that the node has. Thus the connectivity of one node *i *to any other node *j *should approximately follow the rule: .

The ***degree distribution P(k) ***has become one of the most prominent characteristics in network topology. In terms of numerical estimation, a more reliable property, very similar to the previous, is the ***cumulative degree distribution P_c_(k)***. For a power law distribution *P*(*k*) ~ *k*^-γ ^the cumulative degree distribution is of the form *P*(*k*) ~ *k*^(-γ-1) ^and describes the probability of a random chosen node in the network to have a degree *greater *than *k*. Even though lots of research has been done on power law analysis in biological networks, it is still not an established approach widely accepted by the scientific community [101].

To visually represent the properties of the network we usually rank the vertices according to their degree and then plot the degree versus the rank of each vertex. Another representation is to create a histogram by plotting the vertices of the graph sorted according to their degree using a logarithmic scale. A third and very popular representation is to plot the degrees of the nodes sorted versus either their degree distribution *P(k) *or their cumulative degree distribution *P_c_(k)*. An interesting analysis of most of these properties in various PPI, metabolic or transcriptional networks of several organisms (*S. cerevisiae*, *H. pylori*, *C. elegans*) can be found in [100].

A network is called ***assortative ***if the vertices with higher degree have the tendency to connect with other vertices that also have high degree of connectivity; one such category is social networks [102]. If the vertices with higher degree have the tendency to connect with other vertices with low degree then the network is called ***disassortative***. This is characteristic to most molecular interaction networks, where hubs have the tendency to link to nodes with fewer interaction partners rather than to other hubs [103,104]. Newman [102] discusses this property for protein interaction networks, neural networks and food webs.

To correlate the degrees of two nodes *i *and *j *we use a joint probability distribution *P(k_i_, k_j_) = P(k_i_)P(k_j_)*. A more straightforward way is to use the Pearson's Correlation Coefficient (PCC), which quantifies the correlation or linear dependence between two variables (in this case, the degrees of two nodes). In other words, it measures to which extent one variable increases/decreases as the other increases. PCC (*r-value*) between two nodes is defined as the covariance of the two nodes divided by the product of their standard deviations. For the entire network, the assortativity coefficient is the measure of how assortative or disassortative a network is overall. If *M *is the number of edges, and *x_i _*and *y_i _*the degrees of the vertices at either ends of edge *i*, the assortativity coefficient *r *is calculated as follows [102].:

This is equivalent to the Pearson correlation coefficient of the degrees at either ends of an edge. The range of the *r-*values is between *+1 *and -*1, r *<*0 *corresponding to a disassortative network whereas r > 0 to an assortative one. Another way to correlate degrees is to calculate the ***average neighbor degree***. For each vertex *i*, the average degree of its neighbor is calculated as . The values are then averaged for all vertices with the same degree *k*, showing the average neighbor degree *k_nn_(k)*.

## Network Models

Several topological models have been built to describe the global structure of a network, as introduced below.

### Erdös-Rényi model for random graphs [105]

This model was mainly introduced to describe the properties of a random graph. The simple model of a network involves taking a number of vertices *N *and connecting nodes by selecting edges from the *N(N-1)/2 *possible edges randomly. The degree distribution for this model is given by a binomial distribution. The probability of a vertex to have degree *k *is , where 〈*k*〉 is the average connectivity of the network. For small *P *probabilities, the network seems to be disconnected and consists of many isolated components whereas for *P *>*log(N)/N *almost all vertices are connected.

### Watts and Strogatz model [106]

This model was introduced to describe networks that follow the small world topology. This type of topology characterizes many biological networks, like metabolic networks where it often happens that paths of few (three-four) reactions link most metabolites. As a consequence, local changes in metabolite concentration local perturbations in these networks will propagate throughout the entire network. In this model, the frequency of nodes *P(k) *with *k *connections follows a power-law distribution equation *P*(*k*) ~ *k*^-γ^, in which most nodes are connected with small proportion of other nodes and a small proportion of nodes are highly connected. Thus each vertex is connected to *N/2 *nearest neighbors. In exponential networks the probability that a node has a high number of connections is very low.

### Barabasi-Albert model [107]

This model describes scale-free networks and it is one of the most basic models since it describes most of the biological networks [37,108]. The concept behind this model is to reveal information about the dynamics of the network, especially from an evolutionary perspective. The networks are built to mimic gene duplication events, such that they expand continuously by addition of new nodes and the new nodes attach preferentially to sites that are already well connected [109]. Initially we start with small number of nodes *m_0_*. At each step, a new node *m *<*m_0 _*is added and gets linked to the existing network. The probability that a new node is now connected to node *i *is  where *k_i _*is the connectivity of node *i*. The rate of connecting new nodes to node *i *is . The connections are time-dependent so  where *t_i _*is the time point when node *i *enters the network. The probability that a node has degree smaller than *k *is . The probability density of the network is  or , such that the model produces a power law distribution of *γ *= 3.

## Cluster Analysis and Visualization

***Cluster analysis ***[110] aims at classifying a set of observations into two or more mutually exclusive *unknown *groups based on combinations of variables. Thus, cluster analysis is usually presented in the context of *unsupervised *classification [111]. It can be applied to a wide range of biological study cases, such as microarray, sequence and phylogenetic analysis [112]. The purpose of clustering is to group different objects together by observing common properties of elements in a system. In biological networks, this can help identify similar biological entities, like proteins that are homologous in different organisms or that belong to the same complex and genes that are co-expressed [113,114].

It is generally difficult to predict behavior and properties based on observations of behaviors or properties of other elements in the same system, therefore various approaches for cluster analysis emerge. Clustering algorithms may be *Exclusive, Overlapping, Hierarchical *or *Probabilistic*. In the first case, data are grouped in an exclusive way, so that a certain element can be assigned to only one group (exclusively). On the other hand, the overlapping clustering uses fuzzy sets to cluster data, so that each point may belong to two or more clusters with different degrees of membership. A hierarchical clustering algorithm organizes data in hierarchies and is based on the union between the two nearest clusters; it is commonly used for microarray and sequence analysis [115]. A more analytical categorization of clustering algorithms can be found at [110,116].

An important component of a clustering algorithm is the distance measure between data points. If all the components of the data instance vectors have the same physical units, it is then possible that the simple Euclidean distance metric is sufficient to successfully group similar data instances. One example is to cluster cities on a map, since in this case Euclidean distance represents real natural distances. However, for higher dimensional data the Euclidean distance can sometimes be misleading. In that case, a popular measure is the ***Minkowski metric ***and is calculated as  where *D *is the dimensionality of the data. The *Euclidean *can be calculated if we set *p *= 2, while *Manhattan *metric has *p *= 1. There are no general theoretical guidelines for selecting a measure for a given application.

**Figure 10:**
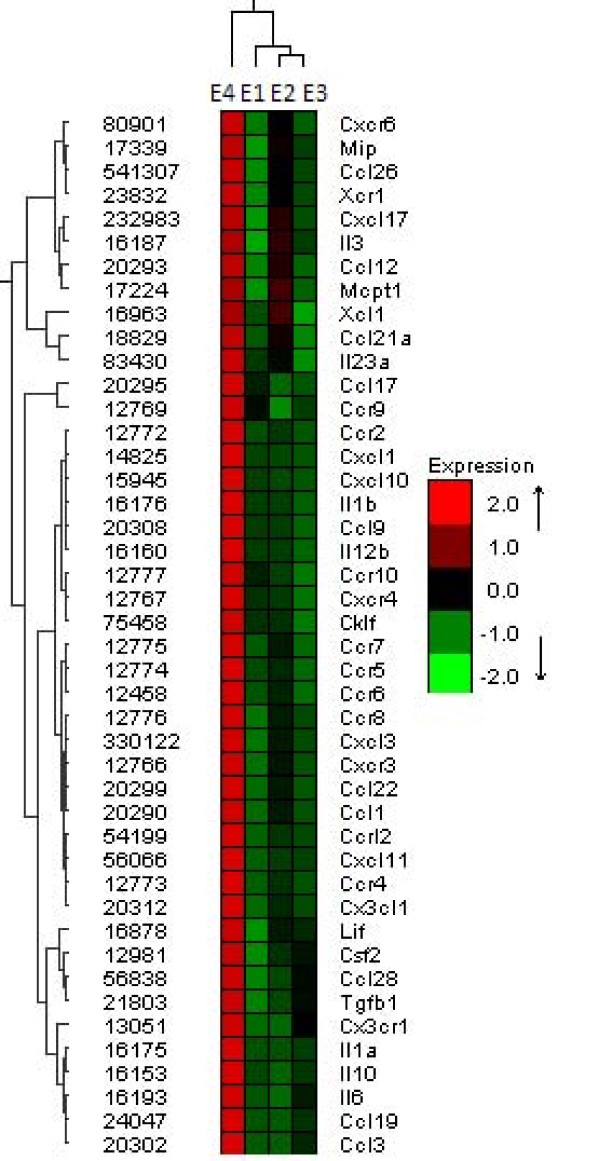
**Average linkage hierarchical clustering example**. The expression of 44 genes was measured in 4 experiments (E_1_, E_2_, E_3_, E_4_). The genes were classified according to their coexpression levels. The Pearson Correlation Coefficient was used (*r-*value) to analyze gene set signal values. Genes were clustered according to the *r-*value correlation matrix using the Average Linkage Hierarchical clustering method. The tree on the left clusters the expressions of the genes whereas the tree on top of the figure clusters the profiles of the experiments. Thus experiments E_2 _and E_3 _are similar and closely related.

**Hierarchical clustering **is a method of cluster analysis which seeks to build a hierarchy of clusters. There are two different strategies to organize data. These are the *agglomerative *and the *divisive*: ***Agglomerative***: It is a "bottom-up" approach. Each observation starts in its own cluster, and pairs of clusters are merged as one moves up the hierarchy. ***Divisive***: This is a "top-down" approach. In this case, all of the observations start by forming one cluster, and then split recursively as one moves down the hierarchy. Some of the most common tree based clustering algorithms that organize data in hierarchies are the Unweighted Pair Group Method with Arithmetic Mean (UPGMA) [117,118], Neighbor Joining [112,119] and Hierarchical Clustering [120,121], all of which represent their clusters as tree structures. The results of hierarchical clustering are usually presented in a dendrogram. Figure 10 shows an example of how genes can be clustered.

Let *n_r _*be the number of clusters and x*_ri _*is the *i*th object in cluster *r *and cluster r is formed from clusters *p *and *q*. In the following, we describe the different methods used to calculate distances between clusters in hierarchical clustering.

***Single linkage ***calculates the smallest distance between objects in the two clusters to merge them: *d*(*r*, *s*) = min(*dist*(*x*_*ri*_, *x*_*sj*_)), *i *∈ (*i*,..., *n*_*r*_), *j *∈ (*1*,....*n*_*s*_).

***Complete linkage ***calculates the largest distance between objects in the two clusters to merge them: *d*(*r*, *s*) = max(*dist*(*x*_*ri*_, *x*_*sj*_)), *i *∈ (*i*,..., *n*_*r*_), *j *∈ (*1*,....*n*_*s*_).

***Average linkage ***uses the average distance between all pairs of objects in any two clusters: . This algorithm is also known as *Unweighted Pair Group Method with Arithmetic Mean (**UPGMA) ***[117,118].

***Centroid linkage ***finds the Euclidean distance between the centroids of the two clusters: , is the Euclidean distance.

***Median linkage ***uses the Euclidean distance between weighted centroids of the two clusters,  are weighted centroids for the clusters *r *and *s*. If cluster *r *was created by combining clusters *p *and *q*, x_r _is defined recursively as .

Single or complete linkages are the fastest of the linkage methods. However, single linkage tends to produce stringy clusters, which is not always preferable. The centroid or average linkage produce better results regarding the accordance between the produced clusters and the structure present in the data. These methods require much more computations. Average linkage and complete linkage may be the preferred methods for microarray data analysis [115].

***Ward's linkage ***finds the incremental sum of squares; that is, the increase in the total within-cluster sum of squares as a result of joining two clusters. The within-cluster sum of squares is defined as the sum of the squares of the distances between all objects in the cluster and the centroid of the cluster. The sum of squares measure is equivalent to the following distance measure ,

where || ||_2 _is the Euclidean distance and  are the centroids of clusters *r *and *s *and *n_r _*and *n_s _*are the number of elements in clusters *r *and *s*.

***Weighted average linkage ***uses a recursive definition for the distance between two clusters. If cluster *r *was created by combining clusters *p *and *q*, the distance between *r *and another cluster *s *is defined as the average of the distance between *p *and *s *and the distance between *q *and *s*: .

***Neighbor Joining ***[112,119] was initially proposed for finding pairs of operational taxonomic units (OTUs) that minimize the total branch length at each stage of clustering of OTUs starting with a star-like tree. The branch lengths as well as the topology of a parsimonious tree can quickly be obtained by using this method [112].

Known platforms that already share the tree-based algorithms described above are the Hierarchical Clustering Explorer (HCE) [122,123], MEGA [124-127] or TM4 [128]. A recent review article shows which file formats, visualization techniques and algorithms can be used for tree analysis [129].

Another category of clustering algorithms tries to cluster data in separate groups by identifying common properties that the nodes of a network share. Different strategies exist, like for example trying to find dense areas in a graph or areas where message exchange between nodes is easier or to identify strongly connected components or clique-like areas etc. Many of such algorithms have been used in different case studies like for example to identify protein families [130], to detect protein complexes in PPI networks [131,132], or for finding patterns and motifs in a sequence [133]. Even though many more exist, some of the most famous algorithms are given below.

***Markov Clustering ***[134] (MCL) algorithm is a fast and scalable unsupervised clustering algorithm based on simulation of stochastic flow in graphs. The MCL algorithm can detect cluster structures in graphs by a mathematical bootstrapping procedure which takes into account the connectivity properties of the underlying network. The process deterministically computes the probabilities of random walks through a graph by alternating two operations: expansion, and inflation of the underlying matrix. The principle behind it is that random walks on a graph are likely to get locked within dense subgraphs rather than move between dense subgraphs via sparse connections. In other words, higher length paths are more often encountered between nodes in the same cluster than between nodes within different clusters, such that the probabilities between nodes in the same complex will typically be higher in expanded matrices. Clusters are identified by alternating expansion and inflation until the graph is partitioned into subsets so that there are no longer paths between these subsets [135,136].

***k*-Means **[137] is a method of cluster analysis which aims to partition *n *observations into *k *clusters in which each observation belongs to the cluster with the nearest mean. K-means and its modifications are widely used for gene expression data analysis [138]. It is a supervised method and users need to predefine the number of clusters. Its complexity is *O(nlk) *where *k *is the number of clusters, *n *the size of the dataset and *l *the loops of the algorithm. The k-means algorithm is one of the simplest and fastest clustering algorithms. However, it has a major drawback: the results of the k-means algorithm may change in successive runs because the initial clusters are chosen randomly.

***Affinity Propagation ***[139] takes as input measures of similarity between pairs of data points and simultaneously considers all data points as potential candidates. Real-valued messages are exchanged between data points until a high-quality set of exemplars and corresponding clusters gradually emerges.

***Restricted Neighborhood Search Cluster Algorithm ***[140]: It tries to find low cost clustering by composing first an initial random clustering. Later it iteratively moves one node from one cluster to another in a random way trying to improve the clustering cost.

**Figure 11:**
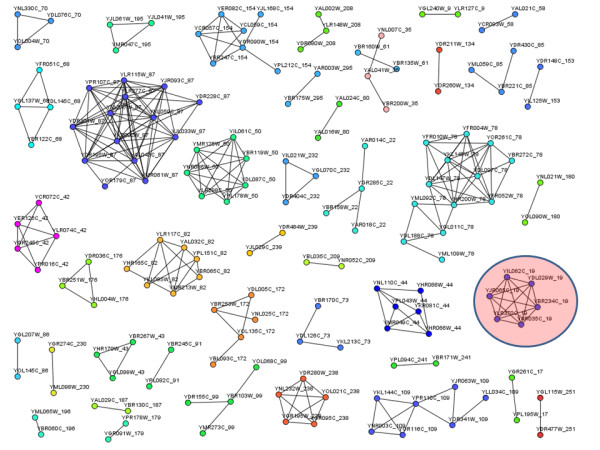
**Predicting protein complexes from PPI networks**. Protein complexes predicted after applying Spectral clustering algorithm and filtering the results in a yeast protein-protein dataset [12] using the jClust application [146]. The budding yeast Arp2/3 complex that is highlighted was successfully predicted.

***Spectral clustering ***[141]: This algorithm tries to find clusters in the graph such that the nodes within a cluster are connected with highly-similar edges and the connections between such areas are weak, constituted by edges with low similarity. The aim is to identify these tightly coupled clusters, and cut the inter-cluster edges. Figure 11 shows an example of protein complex prediction from PPI yeast dataset [12].

Despite the great variety of clustering techniques, many articles directly compare the various clustering methodologies like [135] and [142]. Very often we encounter articles that compare similar algorithms using different datasets and come to very diverse conclusions and results i.e [142,143].

Concerning the visualization of networks, the availability of clustering techniques and their complex configuration/combination, today to a large extent, there is a lack of visualization platforms or tools that are able to integrate a variety of more advanced algorithms and the development implementation of such implementations emerges [144]. Platforms that share clustering algorithms are the Network Analysis Tool (NEAT) [145] and jClust [146] but they are still poor in the variety of methods they offer. Software like ArrayCluster [147] and MCODE [60] is often used in analysis of gene expression profiles and coexpression detection. Many visualization tools [144] such as Medusa [148], Cytoscape [149], Pajek [98] and many others [144] visualize networks in both 2D and 3D, but very few of them like Arena3D [150] try to bridge the gap between clustering analysis and visualization.

## Discussion

**Protein-protein interaction (PPI) networks **[1] are very diverse and it is difficult to come to general conclusions about their properties, mainly because data are generated from different sources both computationally and experimentally as described in a previous section. In most of the cases, PPI networks follow the laws of scale-free networks [93]. In such networks there are always proteins with higher degree of connectivity that appear to be of higher biological significance. Such proteins are the most important for the survival of the cell [93]. Large-scale maps of protein interaction networks have been constructed recently using high-throughput approaches to identify protein interactions [151-155]. It has been shown that these networks are highly dynamic, both during common cellular processes and on the evolutionary scale [109]. Further details on PPI network construction and analysis are given in [156].

**Regulatory networks (GRNs) **are usually sparsely connected. More specifically, the average number of upstream-regulators per gene is less than two [64]. Theoretical results show that the selection for robust gene networks will form minimal complexes even more sparsely connected [64], thus a fundamental design constraint could shape the evolution of gene network complexity. Network maps have been constructed for the transcriptional regulatory networks of *E. coli *and *S. cerevisiae *and are maintained in databases [26,157,158]. They are very sensitive and flexible to evolution [159] since their dynamics changes continuously over time and since transcription factors evolve faster than their target genes [160]. The number of regulators *N_reg _*grows faster than the number of genes *N_tot _*they regulate and it has been shown that  for prokaryotes and  for eukaryotes, where *N *is the network size [161,162]. Mostly they follow the power-law distributions and thus belong to the scale-free network category, even though some of them, like the transcriptional regulatory networks of *E. coli *and *S. cerevisiae *have been shown to possess mixed scale-free and exponential properties [75].

**Signal transduction networks **are characterized by several patterns and motifs like self-sustaining feedback loops. These patterns appear at every time point during the signal transduction in the network and they reveal information about the topology of the network, therefore are important for biological functionality [163]. The nodes with the highest centralities in such networks correspond to domains involved in signal transduction and cell-cell contacts [164]. Signal transduction networks are sparse and they follow the scale-free properties. In *E. coli *and *S. sereviase*, the degree distribution is *P*(*k*) = *k*^-γ^, *γ *≈ 2 and most of the molecules are involved into few interactions and only few of them have higher connectivity [8,165].

**Metabolic and biochemical networks **are scale-free networks indicating a small-world structure considering the topology of the network based on its metabolites [166,167], where all of the nodes in such networks are connected through a short path to any other. One example is presented in [167] for *E. coli*. The probability that a substrate participates as input in *k *metabolic reactions follows the power-law distribution *P*(*k*) = *k*^-*γin*^, *γ_in _*≈ 2.2 whereas the probability of a substrate to be produced by *k *metabolic reactions equals similarly to *P*(*k*) = *k*^-*γout*^, *γ_out _*≈ 2.2. Metabolic networks are extremely heterogeneous and vary from organism to organism. The scale-free structure remains robust even after removal of some central nodes [166] and despite the fact that the architecture of the metabolic networks rests on highly connected substrates [167]. A characteristic feature of these networks is the apparent conservation of network diameter even in distantly related organisms [167]. It has been shown that metabolic networks can form hierarchical structures [168,169] where specific patterns and motifs are overrepresented. Methods to detect such motifs have been applied on network pathways analysis [44,45,47,48], one example being flux mode analysis [48].

## Conclusions

The mathematical discipline which underpins the study of complex networks in biological and other applications is graph theory. It has been successfully applied to the study of biological network topology, from the global perspective of their scale-free, small world, hierarchical nature, to the zoomed-in view of interaction motifs, clusters and modules and the specific interactions between different biomolecules. The structure of biological networks proves to be far away from randomness but rather linked to function. Furthermore, the power of network topology analysis is limited, as it provides a static perspective of what is otherwise a highly dynamic system, such that additional tools should be combined with this approach in order to obtain a deeper understanding of cellular processes.

The complexity of biological networks increases as data are accumulated. The inherent variability of biological data, data inaccuracy and noise, the overload of information and the need to study the dynamics and network topology over time, are currently the bottlenecks in systems biology. Improved techniques for integration of data arising from different sources, as well as for visualization, will be crucial for understanding the functionality of complex networks. Moreover, new mathematical developments in the field and discovery of new areas of applications should be pursued in the near future.

## Competing interests

The authors declare that they have no competing interests.

## Authors' contributions

MS was financially supported by the EMBL PhD Programme. PGB and RS supervised the study. CNM, TGS and SK wrote parts of the manuscript. JA input was crucial for the article. All authors read and approved the final manuscript.
